# Intelligent metasurface imager and recognizer

**DOI:** 10.1038/s41377-019-0209-z

**Published:** 2019-10-21

**Authors:** Lianlin Li, Ya Shuang, Qian Ma, Haoyang Li, Hanting Zhao, Menglin Wei, Che Liu, Chenglong Hao, Cheng-Wei Qiu, Tie Jun Cui

**Affiliations:** 1 State Key Laboratory of Advanced Optical Communication Systems and Networks, Department of Electronics, Peking University, Beijing, 100871 China; 2 State Key Laboratory of Millimeter Waves, Southeast University, Nanjing, 210096 China; 3 Department of Electrical and Computer Engineering, National University of Singapore, 4 Engineering Drive 3, 117583 Singapore, Singapore

**Keywords:** Electronics, photonics and device physics, Optical physics

## Abstract

There is an increasing need to remotely monitor people in daily life using radio-frequency probe signals. However, conventional systems can hardly be deployed in real-world settings since they typically require objects to either deliberately cooperate or carry a wireless active device or identification tag. To accomplish complicated successive tasks using a single device in real time, we propose the simultaneous use of a smart metasurface imager and recognizer, empowered by a network of artificial neural networks (ANNs) for adaptively controlling data flow. Here, three ANNs are employed in an integrated hierarchy, transforming measured microwave data into images of the whole human body, classifying specifically designated spots (hand and chest) within the whole image, and recognizing human hand signs instantly at a Wi-Fi frequency of 2.4 GHz. Instantaneous in situ full-scene imaging and adaptive recognition of hand signs and vital signs of multiple non-cooperative people were experimentally demonstrated. We also show that the proposed intelligent metasurface system works well even when it is passively excited by stray Wi-Fi signals that ubiquitously exist in our daily lives. The reported strategy could open up a new avenue for future smart cities, smart homes, human-device interaction interfaces, health monitoring, and safety screening free of visual privacy issues.

## Machine learning: Metasurfaces gain the power of recognition

Combining radio-frequency imaging with artificial intelligence could make it easier for computers to interact with individuals using non-verbal cues, such as sign language. Lianlin Li from Peking University in Beijing, China and Tie Jun Cui from Southeast University in Nanjing, China, and co-workers fabricated a meter-scale flat panel containing ‘meta-atoms’, tiny electronic devices that manipulate the phases of light waves, arranged in a grid-like pattern. By emitting microwave signals or manipulating stray Wi-Fi signals and detecting echoes bounced back, the metasurface can collect high-resolution imaging data on multiple non-cooperative subjects, even those behind solid walls. The teams fed the microwave data to a series of artificial intelligence algorithms that first identify human shapes, modify signal distributions to better focus on specific body parts, and recognize people's hand signs and vital signs . Experiments showed this setup could continuously monitor hand signals and breathing, even using stray Wi-Fi signals that ubiquitously exist in the daily lives.

## Introduction

The Internet of Things (IoT) and cyber physical systems (CPSs) have opened up possibilities for smart cities and smart homes and are changing the way people live. In this era, there is a strong need to remotely probe where people are, what they are doing, what they want to express by their body language, what their physiological states are, etc., in a way that does not infringe on visual privacy. Recently, developed radio-frequency (RF) sensing technologies have enabled us to realize locating^1,2^ and tracking^3,4^, notable-action recognition^5,6^, human-pose estimation^7,8^, breath monitoring^9,10^, and others^11–13^. These approaches are desirable since they do not require people to carry any active devices or identification tags. However, these systems are typically designed for one specific task and can hardly perform successive tasks adaptively, such as instantly searching for people of interest from a full scene and then adaptively recognizing subtle body features. Furthermore, they are inadequate for monitoring the local body gesture language (e.g., hand signs) and vital signs (e.g., respiration and heartbeat) of human beings in the real world because they require people to be deliberately cooperative. Furthermore, they necessitate weak signals that cannot be reliably distinguished from undesirable disturbances. More importantly, these technologies suffer from complicated system designs and extremely expensive hardware due to the use of a large number of transmitters and/or receivers to extract subtle body information. Thus, it is imperative to develop an inexpensive but intelligent device that can instantly obtain a high-resolution image of a full human body, instantly focus on an arbitrary local body part of interest, and adaptively recognize body signs and vital signs in a smart and real-time way. To realize these demands, we propose the concept of an ANN-driven intelligent metasurface for the adaptive manipulation of electromagnetic (EM) waves, smart data acquisition, and real-time data processing.

The programmable metasurface, as an emerging active member of the metamaterial family^14–23^, is an ultrathin planar array of electronically controlled digital meta-atoms^24–36^. Owing to the unique capability for dynamical and arbitrary manipulations of EM wavefronts, it has elicited many exciting physical phenomena and versatile functional devices, including programmable holography^28^, computational imagers^29–32^, wireless communication systems^33–35^, and others^26,36^. Here, we design a large-aperture programmable metasurface for three purposes in one: (1) to perform in situ high-resolution imaging of multiple people in a full-view scene; (2) to rapidly focus EM fields (including ambient Wi-Fi signals) to selected local spots and avoid undesired interferences from the body trunk and ambient environment; and (3) to monitor the local body signs and vital signs of multiple non-cooperative people in the real world by instantly scanning the local body parts of interest.

Reconstructing a full-scene image, identifying body language, and monitoring human respiration from acquired measurements in real time represent a typical nonlinear EM inverse problem, which is a challenging task due to the inherent time-consuming computations and nonunique solutions. It is also not a trivial issue to model and analyze the characteristics of complicated EM environments (e.g., the indoor environment considered in this work) in a tractable way by using conventional approaches. To overcome these difficulties, we propose a cluster of ANNs, three convolutional neural networks (CNNs), for real-time data processing, which can instantly produce the desired results once they are well trained with a large number of labeled training samples. Due to the ready availability of vast amounts of data and ever-increasing computational power, CNNs have recently been demonstrated to be a powerful tool in various inverse problems^27–47^, including inverse scattering^38–41^, metamaterial design^42–44^, magnetic resonance imaging^45^, and X-ray computed tomography^46^. Our previous results show that CNN-based strategies can remarkably outperform traditional techniques in terms of improved reconstruction quality and reduced computational cost^41^. We establish a synergetic network made of three CNNs, which are end-to-end mappings from microwave data to the desired images and recognition results, and implemented these networks into our intelligent metasurface. In this way, both the global scene and local human-body information can be instantly retrieved.

In this article, we present a proof-of-concept intelligent metasurface working at ~2.4 GHz (the commodity Wi-Fi frequency) to experimentally demonstrate its capabilities in obtaining full-scene images with high resolution and recognizing human-body language and respiration with high accuracy in a smart, real-time and inexpensive way. We experimentally show that our ANN-driven intelligent metasurface works well in the presence of passive stray Wi-Fi signals, in which the programmable metasurface supports adaptive manipulations and smart acquisitions of the stray Wi-Fi signals. This intelligent metasurface introduces a new way to not only “see” what people are doing but also “hear” what people talk without deploying any acoustic sensors, even when multiple people are behind obstacles. In this sense, our strategy could offer a new intelligent interface between humans and devices, which enables devices to remotely sense and recognize more complicated human behaviors with negligible cost.

## Results

**Fig. 1:**
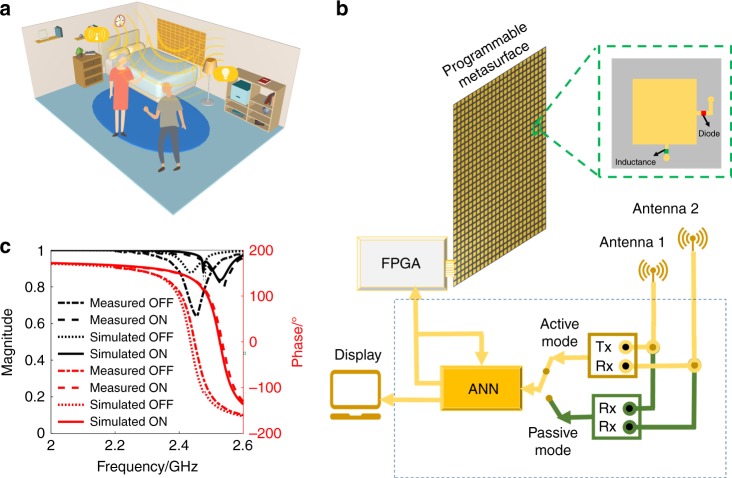
Working principle of the intelligent metasurface. **a** An illustrative scenario for monitoring peoples in a typical indoor environment in a smart, real-time and inexpensive way, where the intelligent metasurface decorated as a part of wall is used to adaptively manipulate ambient Wi-Fi signals. **b** The schematic configuration of intelligent metasurface system by coming a large-aperture programmable metasurface for manipulating and sampling the EM wavefields adaptively with artificial neural networks (ANNs) for controlling and processing the data flow instantly. The intelligent metasurface has two operational modes: active and passive modes. In the active mode, the intelligent system has a transmitting antenna and a receiving antenna. In the passive mode, the intelligent system has a pair of receiving antennas. In addition, the photo of fabricated large-aperture programmable metasurface and the map of meta-atom. **c** Experimental and simulated results of magnitude-frequency and phase-frequency responses of the designed meta-atom

The concept of an ANN-driven intelligent metasurface obtained by integrating a programmable metasurface with deep learning techniques is illustrated in Fig. 1. As shown in Fig. 1b, the designed reflection-type programmable metasurface is composed of 32 × 24 digital meta-atoms with a size of 54 × 54 mm^2^, and each meta-atom is integrated with a PIN diode (SMP1345-079LF) for electronic control. More details on the designed meta-atoms and programmable metasurface are provided in Supplementary Figs. 1, 2. With reference to Fig. 1b, our intelligent metasurface has active and passive modules of operation. In the active module, the metasurface system includes a transmitter (Tx) to emit RF signals into the investigated region through Antenna 1 and a receiver (Rx) to detect the echoes bounced back from the subject through Antenna 2. In the passive module, the system has two or more coherent receivers to collect the stray Wi-Fi waves bounced back from the target subject.

**Fig. 2:**
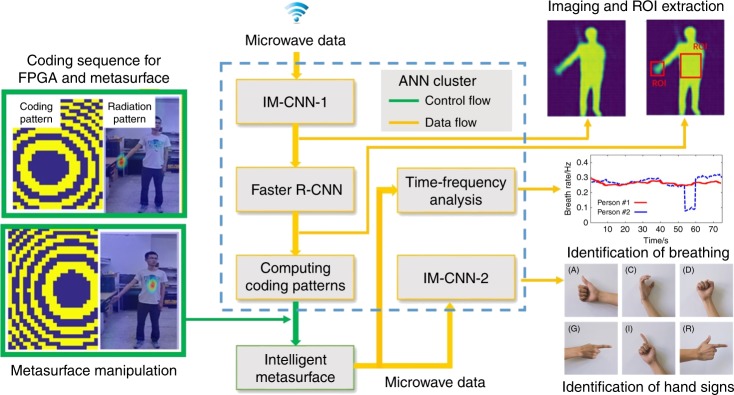
Microwave data processing flow by using deep learning CNN cluster. The microwave data are processed with IM-CNN-1to form the image of the whole human body. Then, the Faster R-CNN is performed to find the region of interest (ROI) from the whole image, for instance, the chest for respiration monitoring, and the hand for sign-language recognition. Afterward, the G-S algorithm is used to find the coding sequence for controlling the programmable metasurface such that its associated radiation beams can be focused toward the desirable spot. IM-CNN-2 processes microwave data to recognize the hand sign; and the human breathing is identified by the time-frequency analysis of microwave data

Figure 2 schematically illustrates three building blocks of the data flow pipeline. In Fig. 2, the microwave data collected by the intelligent metasurface are instantly processed with an imaging CNN (the first CNN of the intelligent metasurface, called IM-CNN-1 for short) to reconstruct the image of the whole human body. More details on IM-CNN-1 are given in the Methods section and Supplementary Fig. 3. Then, a well-developed Faster R-CNN^47^ is adopted to find the region of interest (ROI) within the whole image, for instance, the chest for respiration monitoring and the hand for sign-language recognition. Afterward, a modified Gerchberg-Saxton (G-S) algorithm is implemented to come up with the optimal digital coding sequence for controlling the programmable metasurface so that its radiation wave is focused onto the desired spots, as presented in Supplementary Information. After receiving the command from the host computer, the programmable metasurface will adaptively focus the EM waves onto the desired spots to read the hand signs or physiological state. As such, not only can unwanted disturbances be excluded effectively, but the SNR of echoes from the local body parts of interest can also be remarkably enhanced by a factor of 20 dB, improving the subsequent recognition of hand signs and vital signs (see Supplementary Figs. 6, 7). We develop the other CNN (IM-CNN-2) to process the microwave data to recognize hand signs. In addition, human breath is identified by time-frequency analysis of the microwave data. More details on IM-CNN-2 and the respiration identification algorithm are given in Supplementary Fig. 4. Several sets of representative results are recorded in Supplementary Videos 1, 2.

We first present in situ high-resolution microwave imaging of the whole human body in active mode, which is conducted in our lab environment. In this scenario, the intelligent metasurface system has two horn antennas connected to two ports of the Agilent vector network analyzer (VNA). One antenna is used to transmit EM signals into the investigated domain, and the other receives the EM echoes bounced back from the specimen. In high-resolution imaging, the programmable metasurface serves as a spatial microwave modulator controlled by the field-programmable gate array (FPGA) to register the information about the specimen in a compressive-sensing manner (see Supplementary Information).

To process the microwave data instantly, the *kernel* of the intelligent metasurface for whole-body imaging is IM-CNN-1. To obtain a large number of labeled samples for training IM-CNN-1, a commercial 4-megapixel digital optical camera is embedded in the intelligent metasurface system. The training samples captured by the camera are used to train IM-CNN-1 after being preprocessed with background removal, threshold saturation, and binary-value processing (see Supplementary Fig. 3). The labeled human-body images can be approximately regarded as EM reflection images of the human body over the frequency range from 2.4 to 2.5 GHz. We collect 8 × 10^4^ pairs of labeled training samples in our lab environment, and it takes ~8 h to train IM-CNN-1. The trained IM-CNN-1 can then be used to instantly produce a high-resolution image of the human body in <0.01 s.

**Fig. 3:**
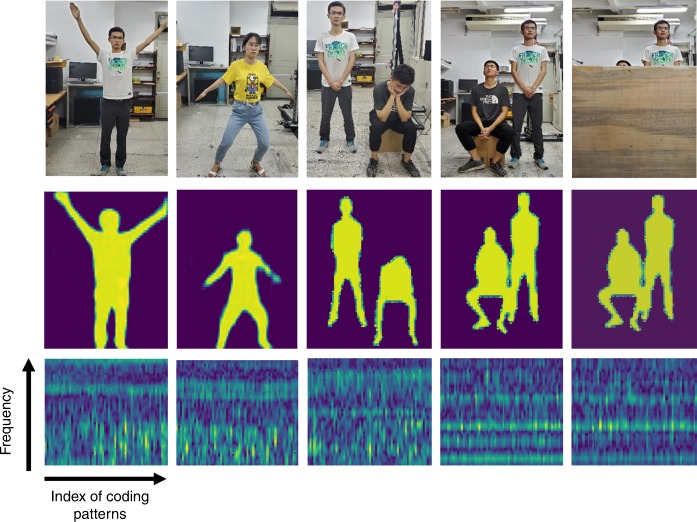
In situ imaging results using the intelligent metasurface with active microwave. (Top row) The first row shows the optical images of specimen, which include single person with different gestures, two persons with different gestures, and two persons behind a 5-cm-thick wooden wall. (Middle) The second row illustrates the corresponding imaging results by the intelligent metasurface with IM-CNN-1. (Bottom) The bottom row presents corresponding amplitudes of microwave data

We experimentally characterize the performance of the intelligent metasurface in obtaining high-resolution images of the whole human body and simultaneously monitoring notable movements in an indoor environment. Two volunteers (coauthors Shuang Ya and Hao Yang Li, referred to as training persons) with different gestures are used to train the intelligent metasurface, while three persons (coauthors Shuang Ya, Hanting Zhao, and Menglin Wei, referred to as testing persons) are invited to test it. The trained intelligent metasurface is then used to produce high-resolution images of the test persons, from which their body gesture information can be readily identified. A series of imaging results are presented in Fig. 3 and Supplementary Video 1. In particular, the “see-through-the-wall” ability of the metasurface is validated by clearly detecting notable movements of the test persons behind a 5-cm-thick wooden wall. Selected results are provided in the rightmost column of Fig. 3, where the corresponding optical images and microwave raw data are given as well. To examine the imaging quality quantitatively, Supplementary Fig. 5a compares the image quality versus the number of random coding patterns of the programmable metasurface in terms of the similarity structure index metric (SSIM)^34^. We show that 53 coding patterns, where 101 frequency points from 2.4 to 2.5 GHz are utilized for each coding pattern, are enough to obtain high-quality images. As reported in the Supplementary Information, the switching time of coding patterns is ~10 μs, implying that the data acquisition time is <0.7 ms in total even if 63 coding patterns are used. Consequently, we safely conclude that the intelligent metasurface integrated with IM-CNN-1 can instantly produce high-quality images of multiple persons in the real world, even when they are behind obstacles.

After obtaining a high-resolution image of the whole body, the intelligent metasurface is then used to recognize the hand signs and vital signs adaptively in real indoor environments. This capacity benefits from the robust feature of the intelligent metasurface in adaptively focusing the EM energy onto the desired spots with very high spatial resolution. This feature supports accurate detection of EM echoes reflected from the human hand for recognizing sign language or from the chest for identifying respiration. Typically, the sign-language rate of the human hand and respiration rate are on the order of 10~30 bps, which is drastically slower than the switching speed of the coding patterns by a factor of 10^5^. Thus, the radiation beams of the intelligent metasurface are manipulated to rapidly scan the local body parts of interest in each observation time interval. As a result, we realize monitoring of the hand signs and respiration of multiple people simultaneously in a time-division multiplexing way (see Supplementary Fig. 4).

**Fig. 4:**
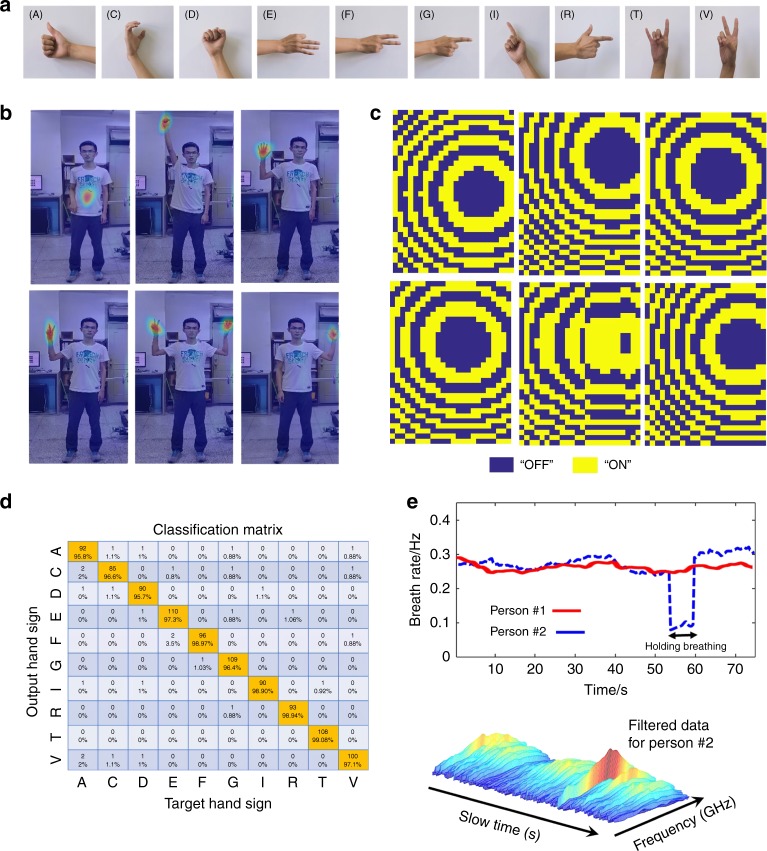
Recognition results of human hand signs and respirations by the intelligent metasurface with active microwave. **a** Ten hand signs of English letters considered in this work. **b**, **c** Selected results of the microwave radiations focused at the desirable spots, for instance, human hands and chest, and corresponding optimized coding patterns of programmable metasurface. In (**b**), the wavefield distributions are obtained using so-called near-field scanning technique (see Supplementary Note 5). **d** The classification matrix of 10 hand signs in (**a**) obtained by using the IM-CNN-2. **e** Results of human respiration of two persons in our lab environment, where person#1 has the normal breathing, and person #2 holds his breathing at around 55 s. From this figure, one can clearly see that not only two states of normal breathing and holding breathing can be readily distinguished, but also the respiration can be accurately identified. In addition, the microwave data with motion filter is also provided

To achieve the complicated task, we propose a three-step routine procedure. First, the Faster R-CNN^47^ is applied to extract the hand or chest part from the full-scene image obtained with IM-CNN-1 in a divide-and-conquer manner. Second, the metasurface is manipulated by adaptively changing its coding pattern to make its radiation beam point to the hand or chest (see Fig. 4a–c). Third, IM-CNN-2, an end-to-end mapping from the microwave data to the label of hand-sign language, is developed to recognize hand signs. Conventional time-frequency analysis is performed for detecting respiration (see Supplementary Fig. 4).

The training samples of IM-CNN-2 include ten hand signs (see Fig. 4a, corresponding to ten different English letters) and 8000 samples for each hand sign. Thus, we have 80,000 samples in total. Figure 4d reports the classification matrix for the ten hand signs with an average recognition accuracy of above 95% by using the intelligent metasurface integrated with IM-CNN-2, where the test people are behind a 5-cm-thick wooden wall. We clearly see that the hand-sign recognition performance is nearly not affected by the number of test persons after the hand parts are well identified by the Faster R-CNN.

Respiration is an important health metric for tracking human physiological states (e.g., sleep, pulmonology, and cardiology). Similar to the recognition of human hand signs, we use the intelligent metasurface to monitor human respiration with high accuracy. Figure 4e reports the results of respiration monitoring of two test persons behind the wood wall. We observe that normal breathing and breath holding are clearly distinguished and that the respiration rate can further be identified with an accuracy of 95% and above, where the ground truth is obtained by a commercial breathing monitoring device. It can be expected that the identification performance is almost independent of the number of test persons due to the use of time-division multiplexing respiration detection.

Our intelligent metasurface works at ~2.4–2.5 GHz, which is exactly the frequency of commodity Wi-Fi signals. Here, we investigate the performance of high-resolution imaging of the full scene and recognition of human hand signs and vital signs when the metasurface is excited by commodity stray Wi-Fi signals. For simplicity, we particularly consider using Wi-Fi beacon signals. In this case, the intelligent metasurface works differently in three major aspects. First, the stray non-cooperative Wi-Fi signals are dynamically manipulated by the metasurface. Second, two or more coherent receiving antennas are used to acquire the Wi-Fi signals bounced back from the subject specimen with the aid of an oscilloscope (Agilent MSO9404A). Third, the microwave data acquired by the receivers are coherently preprocessed before being sent to IM-CNN-1 such that the statistical uncertainties on stray Wi-Fi signals can be calibrated out. More details can be found in Supplementary Video 2 and the Supplementary Information.

**Fig. 5:**
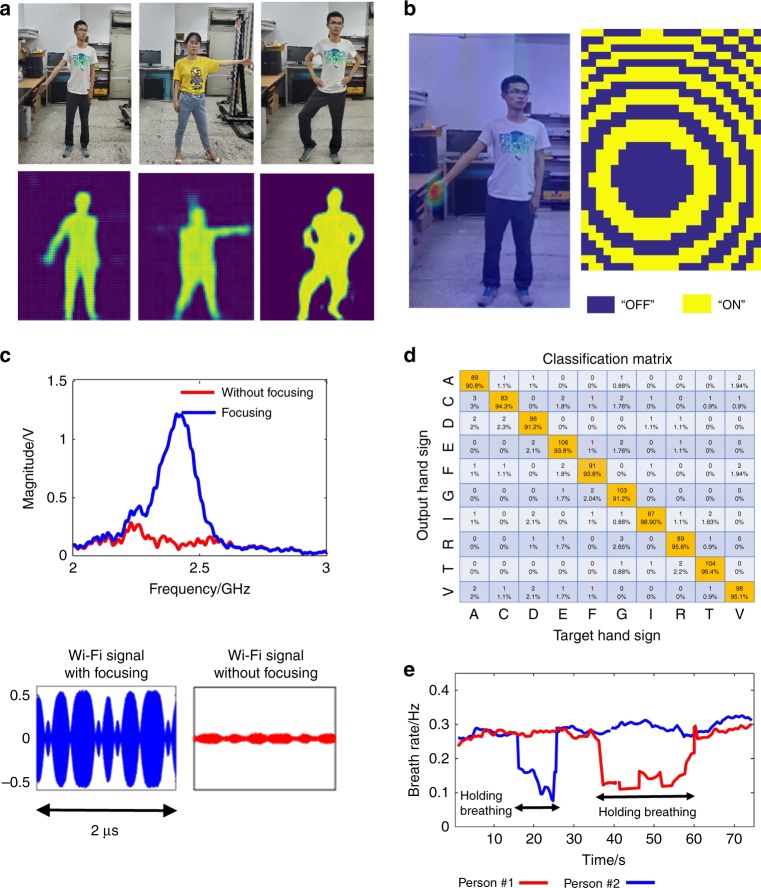
Experimental results of in situ imaging, hand-sign recognition, and respiration identification using the intelligent metasurface in the passive mode with commodity stray Wi-Fi signals. **a** In situ imaging results using the intelligent metasurface excited with commodity Wi-Fi signals. The first row shows the optical images of the subject person with different gestures behind a 5-cm-thick wooden wall. The second row reports corresponding imaging results by the intelligent metasurface with IM-CNN-1. **b** On the left is the result of the Wi-Fi signals focused at the desirable spot of human hand, and on the right is corresponding coding pattern of programmable metasurface. Here, the spatial distribution of Wi-Fi signals is obtained using so-called near-field scanning technique, as done in Fig. 3c. **c** The Wi-Fi signals with and without being focused through the programmable metasurface have been compared, which are measured at the location of left hand shown in (**b**). The top row compares the frequency spectrums of Wi-Fi signals, which are obtained by operating on the raw time-domain Wi-Fi signals with standard FFT technique. Note that the signal-to-noise ratio of Wi-Fi signals at the local spot of human hand can be enhanced by a factor of more than 20 dB at around 2.4 GHz. **d** The classification matrix of 10 hand signs in Fig. 3a obtained by using the IM-CNN-2. **e** Results of human respiration of two non-cooperative persons behind a 5-cm-thickness wall in our lab environment. From this figure, one can clearly see that not only two states of normal breathing and holding breathing can be readily distinguished, but also the respiration can be accurately identified

Figure 5a presents a set of in situ passive imaging results of a subject person behind the wooden wall in our indoor lab environment, where random coding patterns are also used in the programmable metasurface. We surprisingly note that the imaging results obtained by the commodity stray Wi-Fi signals are comparable to those obtained in active mode. Based on the high-resolution images of the full human body, we can realize the recognition of hand signs and vital signs by adaptively performing the routine three-step procedure in active mode. In particular, the Faster R-CNN is operated on the full-scene image to instantly find the location of the hand or chest; then, suitable coding patterns of the intelligent metasurface can be achieved and controlled so that the stray Wi-Fi signals are spatially focused on the desired spots and enhanced; and finally, IM-CNN-2 or the time-frequency analysis algorithm is used to realize the recognition of hand signs and vital signs. As shown in Fig. 5b, c, the commodity Wi-Fi signals can be well focused onto the desired location, e.g., the left hand of the subject person, by using the developed intelligent metasurface. As a result, the SNR of the Wi-Fi signals can be significantly enhanced with a factor of more than 20 dB, which is directly beneficial for the subsequent recognition of hand signs and vital signs (see Supplementary Figs. 7, 8). Figure 5d, e shows the experimental results for hand-sign and respiration recognition of two people, revealing improved accuracies of 90% and 92%, respectively. To summarize, even with illumination by stray Wi-Fi signals, the proposed intelligent metasurface can obtain high-resolution images of a full scene and achieve high-accuracy recognition of hand signs and vital signs of multiple people in a smart and real-time way in the real world.

## Discussion

We devised the concept of an intelligent metasurface imager-cum-recognizer, showing its robust performance in remotely monitoring notable human movements, subtle body gesture language, and physiological states from multiple non-cooperative people in real-world settings. The developed ANN-driven intelligent metasurface relies on two key components: (1) a large-aperture programmable metasurface for adaptive manipulation of EM wavefields and smart data acquisition and (2) three ANNs for smart processing of data flow in real time. We further experimentally demonstrated that the intelligent metasurface works well even when it is passively excited by commodity Wi-Fi signals. This strategy cannot only monitor the notable or non-notable movements of non-cooperative people in the real world but also help people with profound disabilities remotely send commands to devices using body languages. We expect that lip reading and human-mood recognition could also be realized if higher resolution and accuracy are achieved by involving higher frequencies. In principle, the concept of the intelligent metasurface can be extended over the entire EM spectrum, which will open up a new avenue for future smart homes, human-device interaction interfaces, health monitoring, and safety screening.

## Materials and methods

### Design of programmable metasurface

The designed programmable metasurface consists of 32 × 24 meta-atoms operating at ~2.4 GHz, as shown in Supplementary Fig. 1, and the details of the electronically controllable meta-atoms with a size of 54 mm × 54 mm are illustrated in Supplementary Fig. 2. In each meta-atom, a PIN diode (SMP1345-079LF) is integrated to control its EM reflection phase, and the responses of the meta-atom in the ON and OFF states are presented in Fig. 1c. The meta-atom is composed of two substrate layers: the top layer is F4B with a relative permittivity of 2.55 and a loss tangent of 0.0019, and the bottom layer is FR4 with a size of 0.54 × 0.54 mm^2^. The top square patch, integrated with a SMP1345-079LF PIN diode, has a size of 0.37 × 0.37 mm^2^. In addition, a Murata LQW04AN10NH00 inductor with an inductance of 33 nH is used to achieve good separation between the RF and DC signals. CST Microwave Studio is used to design the meta-atom: (1) the reflection response of the meta-atom is investigated under different operation states of the PIN diode; (2) a Floquet port is used to produce an *x*-polarized wave incidence on the metasurface and monitor the reflected wave; and (3) periodic boundary conditions are set on the four sides to model an infinite array.

### Configuration of the intelligent metasurface

The intelligent metasurface has two operational modes: active and passive mode. In active mode, the intelligent system is composed of a large-aperture programmable metasurface, three CNNs, a transmitting antenna, a receiving antenna, and an Agilent VNA. In passive mode, it includes the programmable metasurface, three CNNs, a pair of receiving antennas, and an oscilloscope, in which one antenna serves as a reference receiver to calibrate out the undesirable effects from system error. An optical digital camera, which is used to collect the labeled samples to train the deep ANNs, is synchronized with the whole intelligent metasurface.

The large-aperture programmable metasurface is designed to dynamically and adaptively control ambient EM wavefields by using an FPGA by manipulating its coding sequences, which have a two-fold role. First, it serves as a relay station of information or an electronically controllable random mask, transferring the EM signals carrying finer information about the specimen to the receivers. Second and more importantly, to realize body-language recognition and respiration monitoring, the programmable metasurface with optimized coding patterns can focus the EM wavefields on the desired spots while suppressing the irrelevant interference and clutter.

### IM-CNN-1, IM-CNN-2, and Faster R-CNN

The intelligent metasurface is configured with three deep CNNs for smart and real-time data processing. IM-CNN-1 is designed for converting EM raw data into an image of the whole human body. The Faster R-CNN is a popular classifier originally developed in the area of computer vision^47^ and is used here to identify the hand and chest from the reconstructed whole image. IM-CNN-2 is a classifier used to infer human hand signs from the microwave data.

IM-CNN-1 and IM-CNN-2 operate directly on the microwave raw data, in which the training stage is performed by the ADAM optimization method with a mini-batch size of 32 and 101 epochs. The learning rates are set to 10^−4^ and 10^−5^ for the first two layers and the last layer and halved once the error plateaus. The complex-valued weights and biases are initialized by random weights with a zero-mean Gaussian distribution and a standard deviation of 10^−3^. The training processes are performed on a workstation with an Intel Xeon E5-1620v2 central processing unit, NVIDIA GeForce GTX 1080Ti, and 128GB access memory. The machine learning platform Tensor Flow^48^ is used to design and train the networks in the intelligent metasurface system.

## Supplementary information

Supplementary Materials

SI Video 1

SI Video 2
