# Supplementary material for: Intelligent metasurface imager and recognizer
Source: Light Sci Appl. 2019 Oct 21;8:97. doi: 10.1038/s41377-019-0209-z (PMC6804847; doi:10.1038/s41377-019-0209-z)
Supplement: Supplementary file 1 — Supplementary Materials [file 41377_2019_209_MOESM1_ESM.docx]

**Supplementary Information for**

**Intelligent Metasurface Imager and Recognizer**

Lianlin Li^1+*^, Ya Shuang^1+^, Qian Ma^2^, Haoyang Li^1^, Hanting Zhao^1^, Menglin Wei^1^, Che Liu^2^, Chenglong Hao^3^, Cheng-Wei Qiu^3^, and Tie Jun Cui^2*^

^1^ State Key Laboratory of Advanced Optical Communication Systems and Networks, Department of Electronics, Peking University, Beijing 100871, China

^2^ State Key Laboratory of Millimeter Waves, Southeast University, Nanjing 210096, China

^3^ Department of Electrical and Computer Engineering, National University of Singapore, 4 Engineering Drive 3, Singapore 117583

^+^ These authors contribute equally in this work.

Corresponding authors: [lianlin.li@pku.edu.cn](mailto:lianlin.li@pku.edu.cn); tjcui@seu.edu.cn

**Supplementary Note 1. Design of Meta-Atom**

We elaborate on electronically-controllable binary-phase meta-atom in terms of design parameters, fabrication and tests. As shown in **Supplementary Figure 1(a)**, the meta-atom is composed of two substrate layers: the top substrate is F4B with the relative permittivity of 2.55 and loss tangent of 0.0019, and the bottom substrate is FR4. The top square patch, which is responsible for reflecting incoming electromagnetic (EM) waves, is integrated with a SMP1345-079LF PIN diode connected to the ground plane via a hole. We choose the SMP1345-079LF diode because it has relatively low insertion loss (＜0.2dB) and high isolation>13dB) in the desired frequency band. A TDK chip inductor with inductance L = 33nH (MLK1005S33NJT000) is used to suppress the AC coupling to ground. Geometrical parameters of meta-atom are detailed in **Supplementary Figure 1(b)**.

We examine the EM performance of the electronically-controllable meta-atom numerically and experimentally. In numerical simulations, we use a commercial full-wave EM simulator, CST Microwave Transient Simulation Package 2017. Additionally, a series lumped-parameter circuit is deliberately chosen to model the PIN diode. When the diode is switched ON, it is represented by a 0.7nH inductor in series with a 2Ω resistor. By contrast, when the diode is switched OFF, it is modeled by a 1.8pF capacitor in series with a 0.7nH inductor. The meta-atom has been designed, fabricated and tested. The fabricated sample is shown in the insert of **Supplementary Figure 2(a)**, and the waveguide-based measurement setup is given in **Supplementary Figure 1(c)**, where a standard waveguide to coaxial adapter A-INFO 430WCAS is used. The simulation and experiment results are compared in **Supplementary Figure 1(d)**. We observe that the reflection phase of the meta-atom experiences 180^o^ phase difference when the PIN diode is switched from ON (OFF) to OFF (ON) in the frequency range 2.41-2.48 GHz. The phase change can be accomplished by switching the external DC voltage applied to the PIN diode from 3.3V to 0V.

**Supplementary Note 2. Design of Programmable Metasurface**

The designed programmable metasurface works around 2.42 GHz, consistent with the commodity Wi-Fi frequency. From **Supplementary Figure 2(a)**, the programmable metasurface is composed of independently-controllable 32$\times$24 meta-atoms. Since each meta-atom has a size of 54$\times$54mm^2^, the whole metasurface has size of 1.7$\times$1.3m^2^ in total. **Supplementary Figures 2(a)** and **(b)** show the front and back views of the large-aperture programmable metasurface, respectively. We remark that the whole metasurface is composed of 3$\times4$ identical panels due to the restriction of fabrication, and each panel has 8ⅹ8 meta-atoms.

The whole programmable metasurface is electronically controlled with a FPGA-based Micro-Control-Unit (MCU), as shown in the insert of **Supplementary Figure 2(b)**. A FPGA chip is used to distribute all commands to 768 PIN diodes. To achieve the real-time and flexible controls of 768 PIN diodes soldered in the programmable metasurface, a MCU with size of 90×90mm^2^ is designed and assembled on the upper rear of the metasurface. This MCU is connected with three metasuface panels through three 1.0m-long winding wires in parallel, each of which is connected with another two metasurface panels in series, as illustrated in **Supplementary Figure 2(c)**. The MCU is responsible for dispatching all commands sent from a master computer subject to one common clock (CLK) signal. In our work, the adopted CLK is 50MHz, and the switching time of PIN diode is about 10us each cycle.

From **Supplementary Figure 2(c),** each metasurface panel is equipped with eight 8-bit shift registers (SN74LV595APW), and every 8 PIN diodes share the same shift register. With the use of shift registers, 8 PIN diodes are sequentially controlled. Then MCU will send the commands over 24 independent branch channels, leading to almost real-time manipulations of all PIN diodes. In addition, 768 red-color LEDs are soldiered to indicate the status of the associated PIN diodes, in particular, to indicate clearly whether the PIN diode works well or not. We remark that the proposed control strategy can be readily extended for more PIN diodes by concatenating more metasurface panels, allowing adjustable rearrangement of metasurface panels to meet various needs. In addition, a physical picture of our measurement configuration, when it works in the passive sensing model, has been provided in **Supplementary Figure 2 (d)**.

**Supplementary Note 3. Forward Propagation Model**

The subject target characterized by its reflectivity $O\left( r^{'} \right)$ is located in a complicated indoor environment. We consider two different scenarios: one is for the proposed intelligent metasurface system in the active mode and the other is in the passive mode. Our indoor environment is really complicated, which, besides our experimental instruments, contains desks, desktop computers, metal shelves and objects, chairs, and so on.

**Case 1. Intelligent metasurface in the active mode**

In this scenario, the intelligent metasurface system has a transmitter (Tx) for actively emitting the EM waves into the investigation region through Antenna 1, and a receiver (Rx) for receiving the echoes bounced off the subject specimen through Antenna 2, as shown in **Figure 1**. Additionally, the subject is at the distance of 0.5m~1.0m away from the front side of programmable metasurface. In this case, a VNA is used to emit and acquire microwave signals.

The Tx antenna fixed at $\boldsymbol{r}_{t}$ emits periodically a RF signal denoted by $s(\omega)$, where $\omega$is angular frequency. With such illumination, the programmable metasurface configured with the *m*th coding pattern will give rise to the secondary radiation denoted by $u_{m}(\boldsymbol{r}^{\boldsymbol{'}};\omega)s(\omega)$ ($\boldsymbol{r}^{\boldsymbol{'}}\boldsymbol{\in}\Omega$) inside the investigation region $\Omega$. Under Born assumption, the electrical field acquired by the receiver fixed at $\boldsymbol{r}_{R}$ can be expressed as

$E_{m}\left( \boldsymbol{r}_{R};\omega\right)=s(\omega)\int_{\Omega} G(\boldsymbol{r}_{R},\boldsymbol{r}^{\boldsymbol{'}};\omega)u_{m}(\boldsymbol{r}^{\boldsymbol{'}};\omega)O(\boldsymbol{r'})d\boldsymbol{r}'$, $m=1,2,\ldots, M$ (S1)

For simplicity, we are restricted ourselves into the scalar case. However, the analysis can be readily extended to the full-vector case in a straightforward manner. Here,$M$denotes the total number of coding patterns of metasurface, $G(\boldsymbol{r}_{R},\boldsymbol{r}^{\boldsymbol{'}};\omega)$ denotes the Green’s function of complicated indoor environment. Apparently, $u_{m}(\boldsymbol{r}^{\boldsymbol{'}};\omega)$ heavily depends on the coding patterns, the Green’s function of indoor environment $G(\boldsymbol{r}_{R},\boldsymbol{r}^{\boldsymbol{'}};\omega)$, and other possible parameters of measurement system.

Now, it seems that the reflectivity function $O\left( \boldsymbol{r}^{\boldsymbol{'}} \right)$ can be retrieved from the measurements $\{E_{m}\left( \boldsymbol{r}_{R};\omega\right),m=1,2,\ldots,M\}$ by solving Eq. (1). However, it is not a trivial issue because, for the complicated indoor environment, it remains a challenging open topic to characterize and analyze $G(\boldsymbol{r}_{R},\boldsymbol{r}^{\boldsymbol{'}};\omega)$ and $u_{m}\left( \boldsymbol{r}^{\boldsymbol{'}};\omega\right)$in a tractable manner. Fortunately, deep learning provides a promising tool for tacking such problem due to its unique property of learning the system characteristics from a large amount of training data available. Inspired by this observation, we develop a specialized end-to-end deep learning network, i.e., IM-CNN-1, to solve Eq. (6), which establishes a nearly closed-form solution mapping from the measurements $\left\{ E_{m}\left( \boldsymbol{r}_{R};\omega\right),m=1,2,\ldots,M \right\}$ to the desirable image of $O\left( \boldsymbol{r}^{\boldsymbol{'}} \right)$.

**Case 2. Intelligent metasurface in the passive mode**

In this scenario, the intelligent metasurface illuminated by non-cooperative EM sources will give rise to the secondary EM radiation, and such radiation will further illuminate the subject target. The intelligent metasurface in the passive mode has a pair of coherent receivers (or more coherent receivers), which are connected to two ports of an oscilloscope (Agilent^TM^ MSO9404A) for sampling microwave data.

When illuminated by non-cooperative signal $s_{m}(\omega)$, the intelligent metasurface configured with the *m*th coding pattern within a time interval will give rise to the secondary radiation denoted by $u_{m}\left( \boldsymbol{r}^{\boldsymbol{'}};\omega\right)s_{m}(\omega)$($\boldsymbol{r}^{\boldsymbol{'}}\boldsymbol{\in}\Omega$) inside the investigation domain $\Omega$. Herein, the subscript *m* of $s_{m}(\omega)$ highlights that the non-cooperative Wi-Fi signals varies with the change of coding sequence of the metasurface in an *unknown* way. It is applicable when the data frames of commodity Wi-Fi signals are considered. By contrast, if we only consider the beacon frames of commodity Wi-Fi signals, the unknown signal $s_{m}(\omega)$ is well defined and is nearly unchanged with respect to *m*.

Under the Born approximation, the electrical fields at $\boldsymbol{r}_{1}$and $\boldsymbol{r}_{2}$ scattered from the subject targets are expressed as:

$E_{m}\left( \boldsymbol{r}_{1};\omega\right)=s_{m}(\omega)\int_{\Omega} G_{m}(\boldsymbol{r}_{1},\boldsymbol{r}^{\boldsymbol{'}};\omega)u_{m}(\boldsymbol{r}^{\boldsymbol{'}};\omega)O(\boldsymbol{r'})d\boldsymbol{r}'$ (S2)

and

$E_{m}\left( \boldsymbol{r}_{2};\omega\right)=s_{m}(\omega)\int_{\Omega} G_{m}(\boldsymbol{r}_{2},\boldsymbol{r}^{\boldsymbol{'}};\omega)u_{m}(\boldsymbol{r}^{\boldsymbol{'}};\omega)O(\boldsymbol{r'})d\boldsymbol{r}'$ (S3)

in which$m=1,2,\ldots, M;$ $G_{m}(\cdot,\cdot: \omega)$ is the Green’s function in the indoor environment including the metasurface, and the subscript *m* implies its dependence on the coding pattern of metasurface. In order to calibrate out the unknown signal $s_{m}(\omega)$, we take the correlation average between $E_{m}\left( \boldsymbol{r}_{1};\omega\right)$ and $E_{m}\left( \boldsymbol{r}_{2};\omega\right)$, in particular,

$\left\langle E_{m}\left( \boldsymbol{r}_{1};\omega\right)E_{m}^{*}\left( \boldsymbol{r}_{2};\omega\right) \right\rangle= \left\langle{|s_{m}\left( \omega\right)|}^{2} \right\rangle\int_{\Omega} \int_{\Omega} d\boldsymbol{r}'d\boldsymbol{r}^{''G_{m}}\left( \boldsymbol{r}_{1},\boldsymbol{r}^{\boldsymbol{'}};\omega\right)G_{m}^{*}\left( \boldsymbol{r}_{2},\boldsymbol{r}^{\boldsymbol{'}};\omega\right)u_{m}\left( \boldsymbol{r}^{\boldsymbol{'}};\omega\right)u_{m}^{*}\left( \boldsymbol{r}^{\boldsymbol{''}};\omega\right)O\left( \boldsymbol{r}^{\boldsymbol{'}} \right)O^{*}\left( \boldsymbol{r}^{\boldsymbol{''}} \right)(4)$

where $\left\langle\cdot\right\rangle$ denotes the ensemble average operation. We assume that the non-cooperative Wi-Fi signals and associated radiations are statistically stationary. Thus $\left\langle{|s_{m}\left( \omega\right)|}^{2} \right\rangle$ is almost unchanged with respect to *m*.

Taking above observations into account, we perform the following steps to achieve the estimation of $\left\langle E_{m}\left( \boldsymbol{r}_{1};\omega\right)E_{m}^{*}\left( \boldsymbol{r}_{2};\omega\right) \right\rangle$ for each coding pattern:

- **Step 1**. Acquire a 50$\mu s$-long observation signal for each receiver, and consequently, $y(t,\boldsymbol{r}_{1})$ and $y(t,\boldsymbol{r}_{2})$, for the receivers 1 and 2, respectively.
- **Step 2**. Divide $y(t,\boldsymbol{r}_{1})$ and $y\left( t,\boldsymbol{r}_{2} \right) \mathrm{randomly}$into 200 overlapped 3$\mu s$-long signal samples $\left\{ y^{(i)}\left( t,\boldsymbol{r}_{1} \right), i=1,2,\ldots,200 \right\}$ and $\left\{ y^{(i)}\left( t,\boldsymbol{r}_{2} \right), i=1,2,\ldots,200 \right\}$.
- **Step 3**. Perform FFTs on the above signal samples, i.e.,$FFT\left\{ y^{\left( i \right)}\left( t,\boldsymbol{r}_{1} \right) \right\}, i=1,2,..,200$ and $FFT\left\{ y^{\left( i \right)}\left( t,\boldsymbol{r}_{2} \right) \right\}, i=1,2\ldots200$.
- **Step4**. Calculate the ensemble average

$\left\langle E_{m}\left( \boldsymbol{r}_{1};\omega\right)E_{m}^{*}\left( \boldsymbol{r}_{2};\omega\right) \right\rangle=\frac{1}{200}\sum_{i=1}^{200} {FFT\left\{ y^{\left( i \right)}\left( t,\boldsymbol{r}_{2} \right) \right\}\left[ FFT\left\{ y^{\left( i \right)}\left( t,\boldsymbol{r}_{2} \right) \right\} \right]}^{*}$.

If we consider the indoor environment except the intelligent metasurface as a random process, then Eq. (4) can be expressed as

$$\left\langle E_{m}\left( \boldsymbol{r}_{1};\omega\right)E_{m}^{*}\left( \boldsymbol{r}_{2};\omega\right) \right\rangle=$$

$$\left\langle{|s_{m}\left( \omega\right)|}^{2} \right\rangle\int_{\Omega} \int_{\Omega} d\boldsymbol{r}'d\boldsymbol{r}''\left\langle G_{m}(\boldsymbol{r}_{1},\boldsymbol{r}^{\boldsymbol{'}};\omega)G_{m}^{*}(\boldsymbol{r}_{2},\boldsymbol{r}^{\boldsymbol{'}};\omega) \right\rangle\left\langle u_{m}(\boldsymbol{r}^{\boldsymbol{'}};\omega)u_{m}^{*}(\boldsymbol{r}^{\boldsymbol{''}};\omega) \right\rangle O(\boldsymbol{r'})O^{*}(\boldsymbol{r''})$$

$m=1,2,\ldots, M$ (S5)

For simplicity, we take numerical discretion of the investigation domain $\Omega$, so that the following approximations hold

$\left\langle u_{m}(\boldsymbol{r}^{\boldsymbol{'}};\omega)u_{m}^{*}(\boldsymbol{r}^{\boldsymbol{''}};\omega) \right\rangle\approx\left\langle\left| u_{m}\left( \boldsymbol{r}^{\boldsymbol{'}};\omega\right) \right|^{2} \right\rangle\delta(\boldsymbol{r-r'})$ (S6)

and/or $\left\langle G_{m}(\boldsymbol{r}_{1},\boldsymbol{r}^{\boldsymbol{'}};\omega)G_{m}^{*}(\boldsymbol{r}_{2},\boldsymbol{r}^{\boldsymbol{'}};\omega) \right\rangle\approx\left\langle\left| G_{m}(\boldsymbol{r}_{1},\boldsymbol{r}^{\boldsymbol{'}};\omega) \right|^{2} \right\rangle\delta(\boldsymbol{r-r'})$ (S7)

Note that Eq. (7) can be justified in terms of the time-reversal theory in random surrounding media or cavity. As a consequence, Eq. (5) becomes

$$\left\langle E_{m}\left( \boldsymbol{r}_{1};\omega\right)E_{m}^{*}\left( \boldsymbol{r}_{2};\omega\right) \right\rangle=\left\langle{|s_{m}\left( \omega\right)|}^{2} \right\rangle\int_{\Omega} d\boldsymbol{r}'\left\langle\left| G_{m}(\boldsymbol{r}_{1},\boldsymbol{r}^{\boldsymbol{'}};\omega) \right|^{2} \right\rangle\left\langle\left| u_{m}\left( \boldsymbol{r}^{\boldsymbol{'}};\omega\right) \right|^{2} \right\rangle\left\langle\left| O\left( \boldsymbol{r}^{\boldsymbol{'}} \right) \right|^{2} \right\rangle(S8)$$

We observe from Eq. (5) or Eq. (8) that the coherent measurements between a pair of receivers $\{\left\langle E_{m}\left( \boldsymbol{r}_{1};\omega\right)E_{m}^{*}\left( \boldsymbol{r}_{2};\omega\right) \right\rangle, m=1,2,\ldots,M)$ behave as a function of the reflectivity of subject target $O\left( \boldsymbol{r}^{\boldsymbol{'}} \right)$. Although the relation cannot be analyzed or tackled in an analytical way, the deep learning technique can be utilized to retrieve the reflectivity function $O\left( \boldsymbol{r}^{\boldsymbol{'}} \right)$ from coherent measurements $\{\left\langle E_{m}\left( \boldsymbol{r}_{1};\omega\right)E_{m}^{*}\left( \boldsymbol{r}_{2};\omega\right) \right\rangle, m=1,2,\ldots, M\}$.

**Supplementary Note 4. IM-CNN-1, IM-CNN-2, Respiration Identification Algorithm**

The intelligent metasurface is configured with three deep ANNs for data processing. IM-CNN-1 is designed for transferring the microwave data into the image of the whole human body. The Faster R-CNN [47] is a classifier to identify the local body region (e.g. hand and chest) from the whole image transferred from IM-CNN-1. IM-CNN-2 is a classifier to recognize the hand signs from microwave data, where the radiation beam of programmable metasurface has been manipulated to focus on the hand of interest.

**1. Design and training of IM-CNN-1 and IM-CNN-2**

IM-CNN-1 is an end-to-end mapping from the complex-valued microwave data to the desired images, which has been schematically shown in **Supplementary Figure 3(b)**. Similarly, IM-CNN-2 is shown in **Supplementary Figure 3(c)**, where the input is complex-valued microwave signal, and the output is the recognized label of hand signs. To improve the stability of artificial neural network, and meanwhile avoid the so-called gradient exploding and vanish, the proposed IM-CNN-1 and IM-CNN-2 are composed of a cascade of residual CNNs. In these figures, BN denotes the batch normalization, Softmax denotes soft-max nonlinear activation function, $k\left( a,b,c \right)$ denotes the convolutional kernel with size of $a\times b\times c$, and $n\left( a \right)$ denotes the number of convolutional kernels to be $a$.

The training procedure of IM-CNN-1 is briefly outlined in **Supplementary Figure 3(a)**. A commercial 4-megapixel digital optical camera is embedded in the intelligent metasurface system to obtain a large amount of labeled samples as calibrations for training IM-CNN-1. The labeled human-body images captured by the camera after background removal and binarization processing can be approximately regarded as the microwave reflectivity images of the human body, because the microwave reflection of the human body can be approximated to be homogenous over the undergoing frequencies from 2.4 to 2.5GHz.

The training stage is done using the ADAM optimization method, with mini-batches size of 32, and epoch setting as 50. The learning rates are set to 10^−4^ and 10^−5^ for the first two layers and the last layer in each network, respectively, and halved once the error plateaus. The complex-valued weights and biases are initialized by random weights with zero-mean Gaussian distribution and standard deviation of 10^−3^. The computations are performed with AMD Ryzen Threadripper 1950X 16-Core processor, NVIDIA GeForce GTX 1080Ti, and 128GB access memory. The networks are designed using the Tensor Flow library.

**2. Respiration identification algorithm**

We explain the principle behind the proposed respiration identification algorithm by considering an example with a quasi-stationary subject. The mechanical movement of lung produce a chest surface vibration, which provides the feasibility for respiration identification. Such vibration will cause a Doppler frequency on the transmitted EM waveform. Depending on the subject activity and health condition, this frequency is within the range 0.1~0.3 Hz. The Doppler frequency carrying the respiration information can be readily detected by using our intelligent metasurface. For easy discussions, we introduce several necessary notations. To detect the respiration of the subject, we periodically acquire the echoes bounced from the human chest every $T_{p}$ seconds, and each acquired echo has duration of $T_{d}$ seconds. Apparently, $T_{d}<T_{p}$. As such, we obtain $N_{b}$ echoes in slow-time domain.

With support of **Supplementary Figure 4**, we give the details of algorithm for respiration extraction as following. Firstly, a data matrix $\mathbf{M}$ with $N_{b}$ columns is arranged, in which each column corresponds to a $N_{d}$-length signal, where $N_{d}$ denotes the number of discrete frequency points. Consequently, the data matrix $\mathbf{M}$ has the size of $N_{d}\times N_{b}$. Secondly, the well-known second-order motion filter is applied row-by-row to $\mathbf{M}$**,** so that the unwanted background signals can be readily filtered out. Thirdly, the classical shot-time FFT is performed by sliding the window along the slow time domain, and then the respiration information can be easily estimated by picking up the Doppler frequency component with maximum intensity.

Above discussions is valid to the respiration detection of one subject, but such strategy can be readily extended for monitoring multiple subjects at different azimuth directions by exploring the so-called time-division multiplexing technique, as shown in **Supplementary Figure 4(a)**. Since the human respiration period is overwhelmingly longer than that of the switching time of intelligent metasurface by a factor of about ${10}^{3}\sim{10}^{5}$, multiple subjects can be rapidly scanned within one observation interval $T_{d}$in time-division multiplexing manner. In particular, the EM beams of intelligent metasurface are successively and rapidly pointed towards different subjects within one observation interval $T_{d}$, since the locations of human body chests have been detected in high resolution. In this way, the respiration information of multiple non-cooperative people can be monitored almost simultaneously. Here, we would like to emphasize that such time-division multiplexing operation mechanism is also applicable to the recognition of hand signs of multiple non-cooperative persons.

**Supplementary Note 5. Modified G-S algorithm**

Here, we provide the details to achieve the coding sequence (or coding pattern) of programmable metasurface, so that the associated radiation beam can be adaptively pointed towards the desired spot. Although many efforts have been made to optimize the coding sequence of metasurface, most of them focus on tailoring the far-field radiation. However, in this study, the subject specimen falls into the near-field region of metasurface. Hence we need to consider the dynamic manipulation of EM near-field radiation by controlling the programmable metasurface. To this end, we make two-aspect efforts. Firstly, the radiation field of meta-atoms has been modeled in term of the well-known Huygens’ principle, by which more realistic EM interactions between the meta-atom and EM wavefield can be fully taken into account. Secondly, the total scattering field from the whole coding metasurface aperture is synthesized by the superposition principle.

In light of the Huygens’ principle, the EM response of meta-atom can be accurately obtained once its induced equivalent current is known. Taking this fact into account, we start by evenly dividing the meta-atom into *P*$\times Q$ grids with subwavelength scale, and then the induced current over each grid is approximated uniform. The relationship between the scattering field **E** of each meta atom and induced equivalent current **J** can be represented as

$\mathbf{E}\left( \mathbf{r} \right)=\sum_{p_{x}=1}^{P} \sum_{p_{y}=1}^{Q} i\omega\mu\int_{\Delta} \bar{\mathbf{G}}\left( \mathbf{r},\mathbf{r}_{p_{x},p_{y}}^{'}+\delta\mathbf{r}^{'} \right)\cdot\mathbf{J}\left( \mathbf{r}_{p_{x},p_{y}}^{'}+\delta\mathbf{r}^{'} \right) d{\delta\mathbf{r}}^{'}$ (S9)

where $\bar{G}\left( \mathbf{r},\mathbf{r}^{\boldsymbol{'}} \right)=\left[ \bar{I}+\frac{\nabla\nabla}{k_{0}^{2}} \right]\frac{e^{{jk}_{0}\left| \mathbf{r}-\mathbf{r}' \right|}}{4\pi\left| \mathbf{r}-\mathbf{r}' \right|}$ is the dyadic Green's function in free space, $\mathbf{r}_{p_{x},p_{y}}^{'}$ denotes the central coordinate of the$\left( p_{x},p_{y} \right)^{th}$grid, and $k_{0}$ is wavenumber. The integration in Eq. (9) is implemented over the grids of meta-atom whose area is $\Delta$. In the limit of $\Delta\to0$, Eq. (9) becomes $i\omega\mu\Delta\sum_{p_{x}=1}^{P} \sum_{p_{y}=1}^{Q} \bar{G}\left( \mathbf{r},\mathbf{r}_{p_{x},p_{y}}^{'} \right)\cdot\mathbf{J}\left( \mathbf{r}_{p_{x},p_{y}}^{'} \right)$, which is exactly the one used in tailoring the far-field radiation. For numerical implementation, Eq. (9) can be reformulated in a compact form

$$\mathbf{E}=\left[ \begin{aligned} E_{\mathbf{r}}^{x} \\ E_{\mathbf{r}}^{y} \end{aligned} \right]=\left[ \begin{matrix} A_{\mathbf{r},\mathbf{r}'}^{xx} & A_{\mathbf{r},\mathbf{r}'}^{xy} \\ A_{\mathbf{r},\mathbf{r}'}^{yx} & A_{\mathbf{r},\mathbf{r}'}^{yy} \end{matrix} \right]\left[ \begin{matrix} J_{\mathbf{r}'}^{x} \\ J_{\mathbf{r}'}^{y} \end{matrix} \right]=\mathbf{AJ} (S10)$$

where $\mathbf{r}'$ denotes the central coordinate of all divided grids, $\mathbf{r}$ denotes the observation position, **A** is a mapping matrix with entries coming from the Dyadic Green’s function, and the induced current is organized into a $2\times P\times Q$ column vector **J**. Then, an element model either at ON or OFF state illuminated by a plane wave is calculated through numerical simulation, and the resultant co-polarized scattering field is collected at a distance far enough from the element, arranged into a column vector **E**. The least-square method is used to retrieve the induced equivalent current **J**:

$\mathbf{J}=\left( \boldsymbol{A}^{\boldsymbol{'}}\boldsymbol{A}+\gamma\boldsymbol{I} \right)^{+}\boldsymbol{A}^{'}\mathbf{E} (S11)$

where $\gamma$ denotes an artificial regularization parameter, ***I*** means the unit matrix, and + presents the matrix pseudo inverse. In our specific implementations, the surface of meta atom is uniformly divided to 10×10 square grids with area of 0.044λ×0.044λ, and the scattering field **E** is obtained at the distance of 0.5λ (λ denotes operational wavelength) away from the meta-atom illuminated by a plane wave with intensity of 1V/m. The equivalent induced current of meta-atom at 2.45GHz can be obtained according to Eq. (11), where $\gamma={10}^{10}$ is used.

We next synthesize the EM wavefield induced from the whole programmable metasurface in terms of the superposition principle. For simplicity, the electrical field from a single EM source (either a cooperative active source or a non-cooperative Wi-Fi router) is considered, which can be approximated as a typical spherical wave, i.e.,

$$E^{in}=\left| E^{in} \right|\exp\left( j\varphi_{in} \right)=A_{0} {cos}^{q}\left( \theta_{f} \right)\frac{\exp\left( jk_{0}r_{f} \right)}{r_{f}} (S12)$$

where *A*_0_ is a calibration constant, $\theta_{f} \mathrm{and}r_{f}$ denote the elevation angle and the observation distance in the spherical coordinate system, respectively. Here, the EM interaction among different meta-atoms have been ignored due to the relatively big distance among meta-atoms of being around half operation wavelength.

Finally, we can achieve the flowchart of modified G-S algorithm procedure as summarized in **Supplementary Figure 5(a)**. Mathematically, the G-S algorithm is performed to minimize the following objective function, i.e.,

$obj=\sum_{i}^{p_{x}} \sum_{j}^{p_{y}} {(E_{goal}\left( i,j \right)-\left| E_{s}\left( i,j \right)+E_{in}(i,j) \right|)}^{2} \left( S13 \right)$

where $E_{goal}$ represents the desired spatial intensity distribution in observation plane. Apparently, such solution strategy behaves in an iterative way. As for its initial guess, the meta atoms of programmable metasurface are randomly set to be ON or OFF state. After a few tens of iterations in several seconds, the stable convergence can be achieved.

A set of simulation and experimental results are provided to verify the proposed methods. To this end, a transmitting horn antenna located at (0, -0.3m, 0.8m) is used to illuminate the programmable metasurface, and its resultant radiation is received by an open-ended waveguide probe. Both transmitting antenna and receiving waveguide probe are connected to two ports of a vector network analyzer. In our implementation of near-field scanning, a standard waveguide probe is mechanically scanned within a square area of 1.9m×1.5m square with sampling space of 0.03m. We consider six different coding sequences of programmable metasurface, as shown in the top row of **Supplementary Figure 5(b)**. Accordingly, the simulated spatial distributions of EM radiations at three observation planes of z=1.0, 1.3, and 1.5m away from the front side of programmable metasurface are reported in the second to fourth rows of this figure, respectively. Here, simulation results are obtained by using the CST-based full-wave simulations. For comparison, experimental results on the observation plane of z=1.5 m are provided in the bottom row of **Supplementary Figure 5(b)**. Note that in these figures, the electrical field distributions have been normalized by their own maximums. It is clear from our results that the experimental results agree with simulation results very well.

Finally, some remarks on the modified GS algorithm are listed. Note that the modified GS algorithm described above is based on the free-space Green function $\bar{G}\left( \mathbf{r},\mathbf{r}^{\boldsymbol{'}} \right)$. As a result, in principle, the focused point will be blurred and derived from the desired point due to so-called multiple scattering effect, when the modified GS algorithm is directly applied for treating the complex environment. Fortunately, it can be observed from our experimental results provided in **Supplementary Figure 5** that, the focus error can be ignored for our lab environment. Of course, if the indoor environment is so complex that such that the error cannot be ignored, some mitigation techniques need to be applied. For instance, the ray-tracing based auto-focusing technique widely utilized in the area of penetration radar imaging could be used to remove the unwanted effect from the wall. Further, deep learning techniques could be employed to learn the real Green’s function of unknown surrounding environment. Once the real Green’s function is learned, the modified GS algorithm described above can be generalized for treating the complex surrounding environment in a straightforward manner.


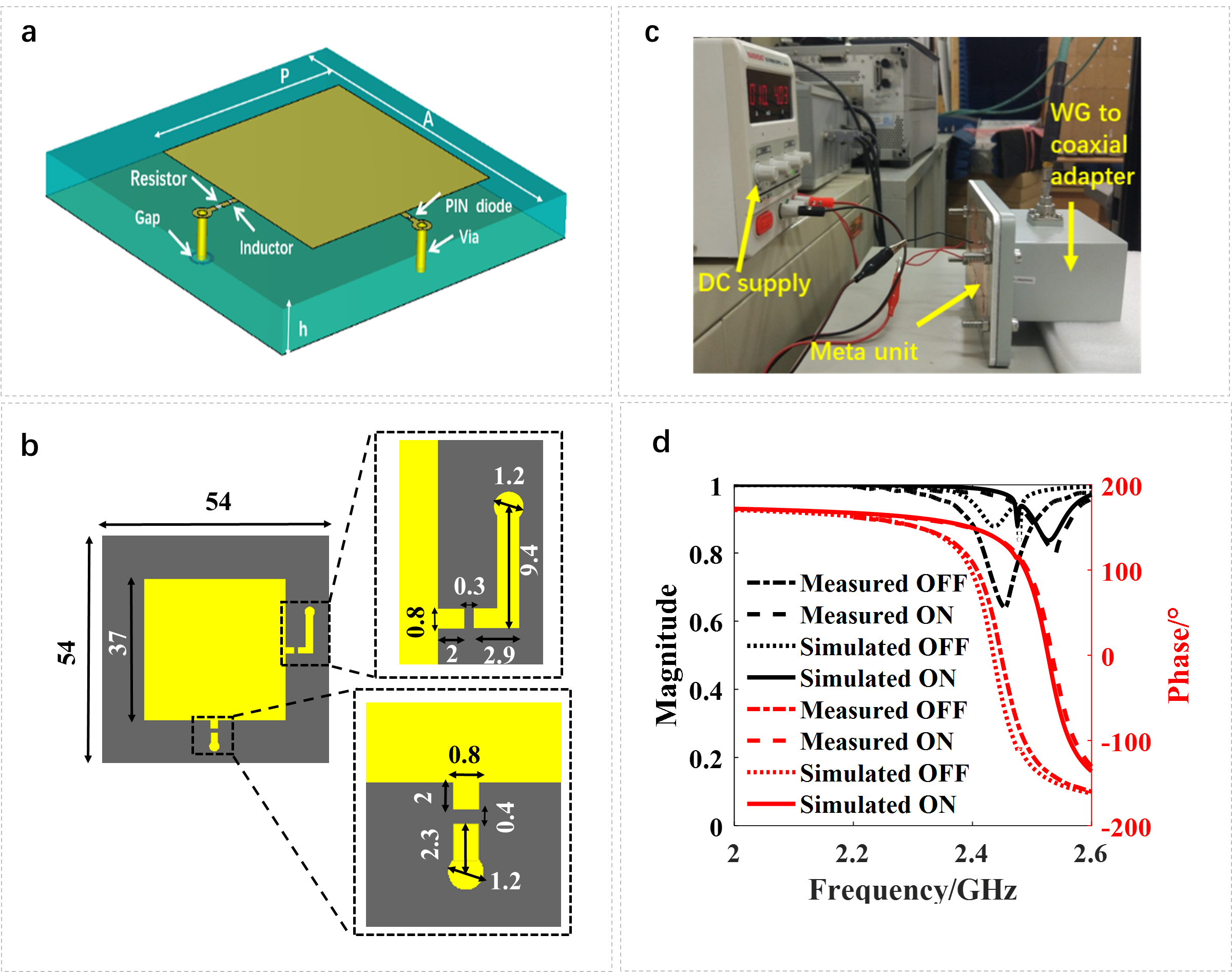


**Supplementary Figure 1** | **Design and test of electronically-controllable meta-atom**. **(a)** the sketched map of designed meta-atom. **(b)** the details of geometrical parameters of designed meta-atom, where the unit is mm. **(c)** the waveguide-based experimental setup of the designed meta-atom. **(d)** experimental and simulated results of magnitude-frequency and phase-frequency responses of the designed meta-atom.


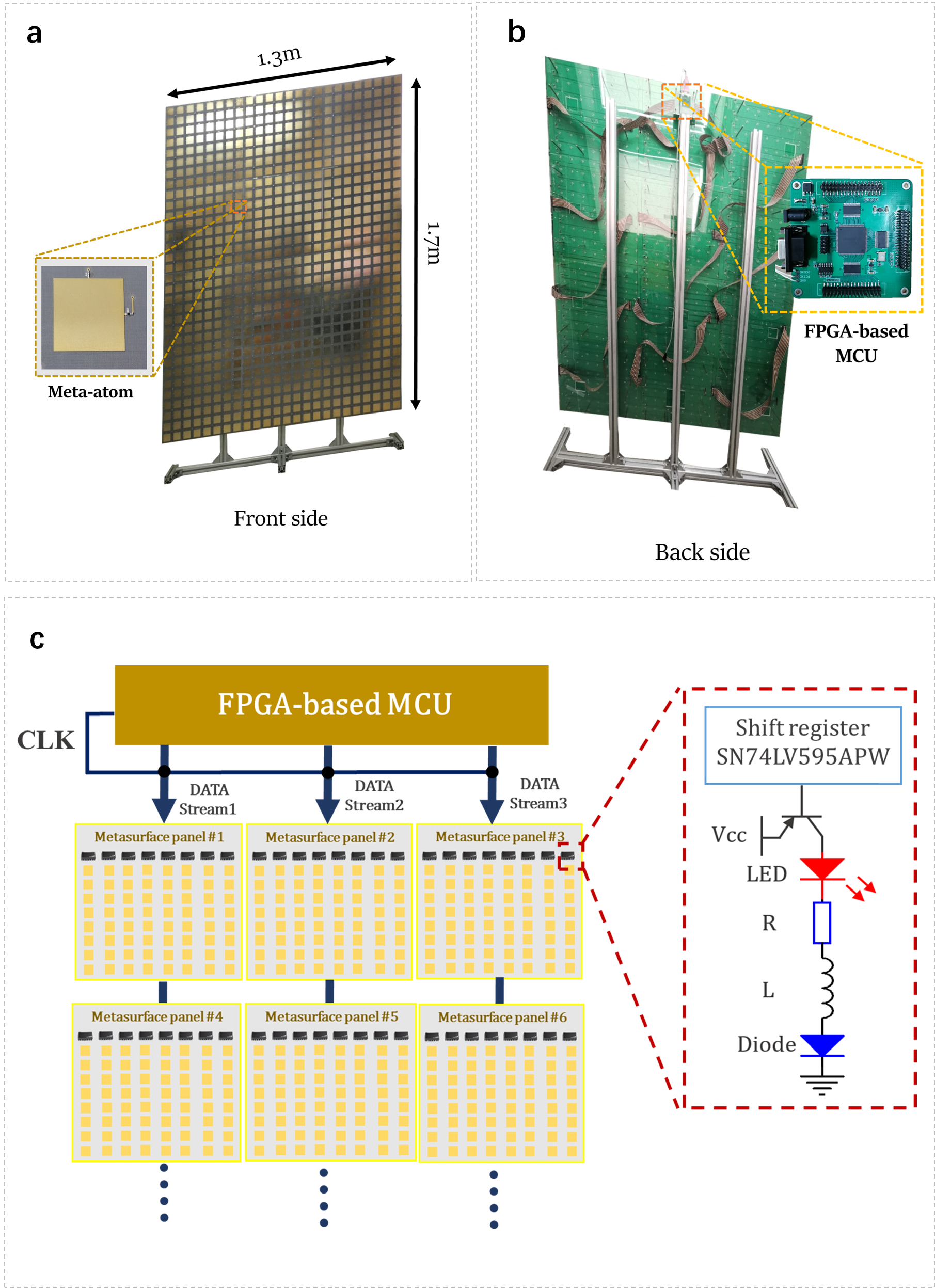


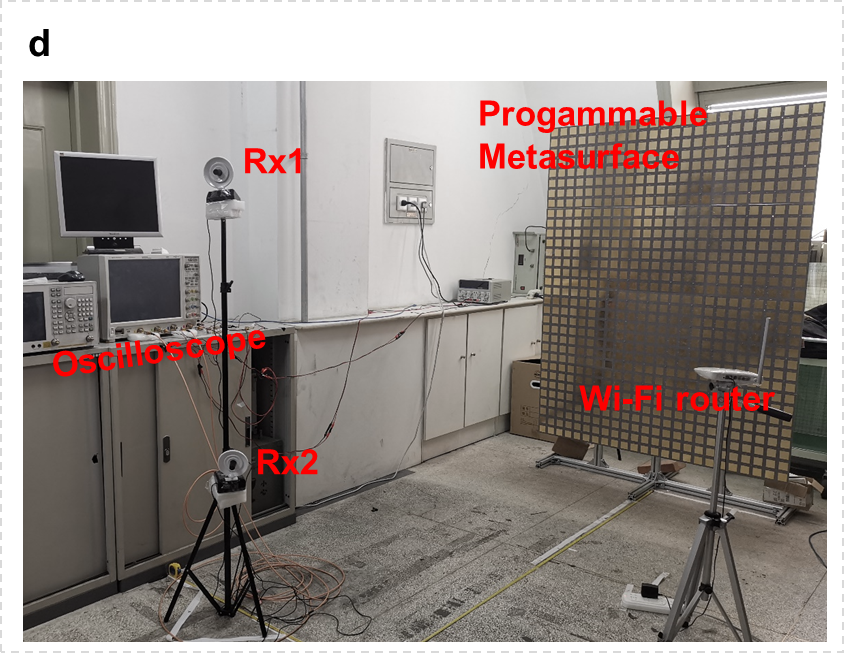


**Supplementary Figure 2 | Designed large-aperture programmable metasurface and its controlling scheme. (a)-(b)** The pictures of the designed programmable metasurface with size of 1.3ⅹ1.7m^2^, where the front- and back-view pictures are provided. In this set of figures, the designed meta-atom and FPGA-based micro control unit (MCU) are inserted in **a** and **b**, respectively. **(c)** The control architecture of the FPGA-based MCU and zoomed version of logical circuit on the metasurface panel. (**d**)The physical picture of measurement configuration at the passive mode. Note that when our intelligent sensing system works at the passive mode, there are two receiving antennas, Rx1 and Rx2, which are connected to an oscilloscope. In contrast, if the sensing system works at the active mode, Rx 1 is used for the transmitter, and these two horn antennas are connected to the VNA instead of oscilloscope.


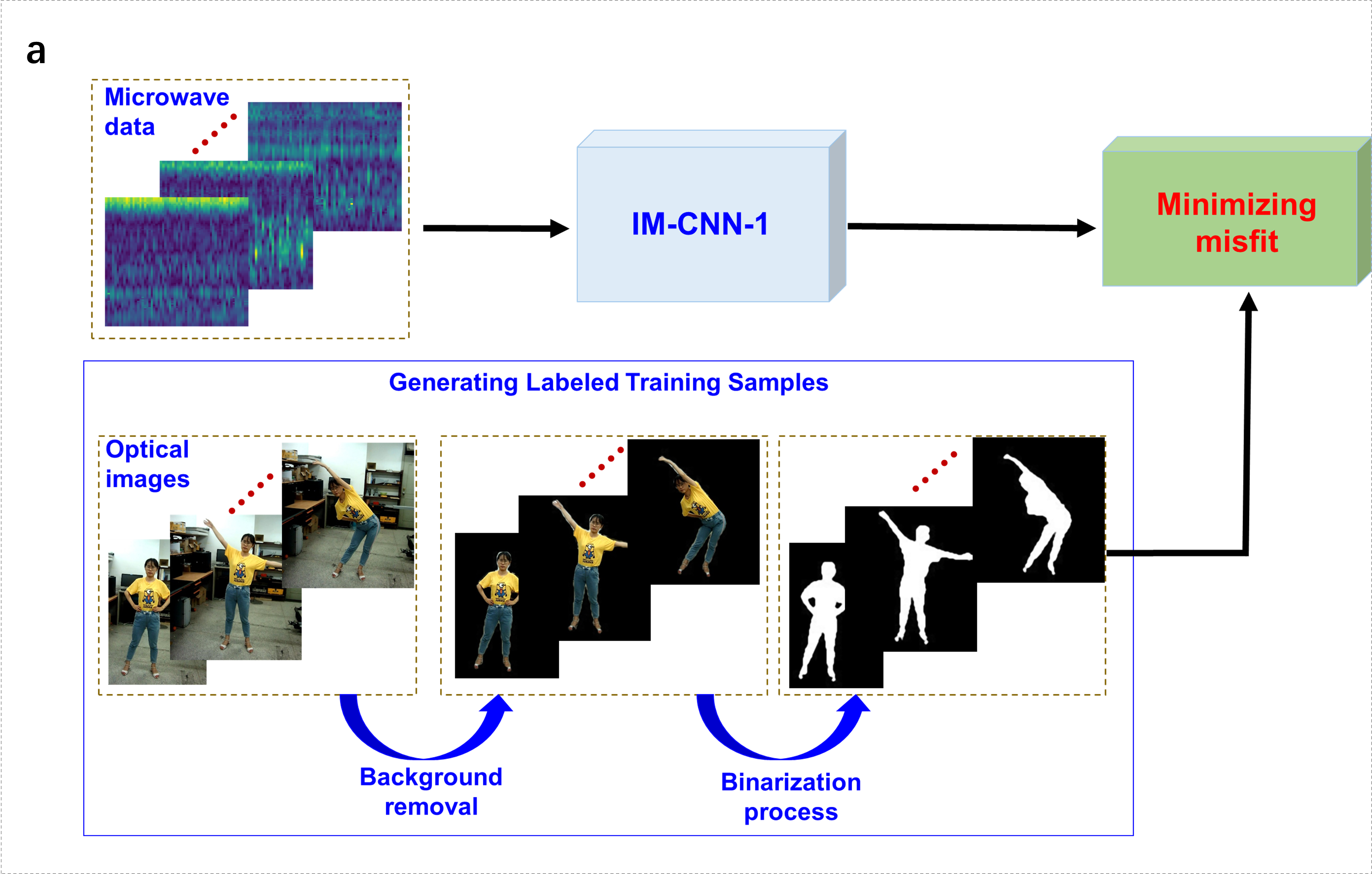


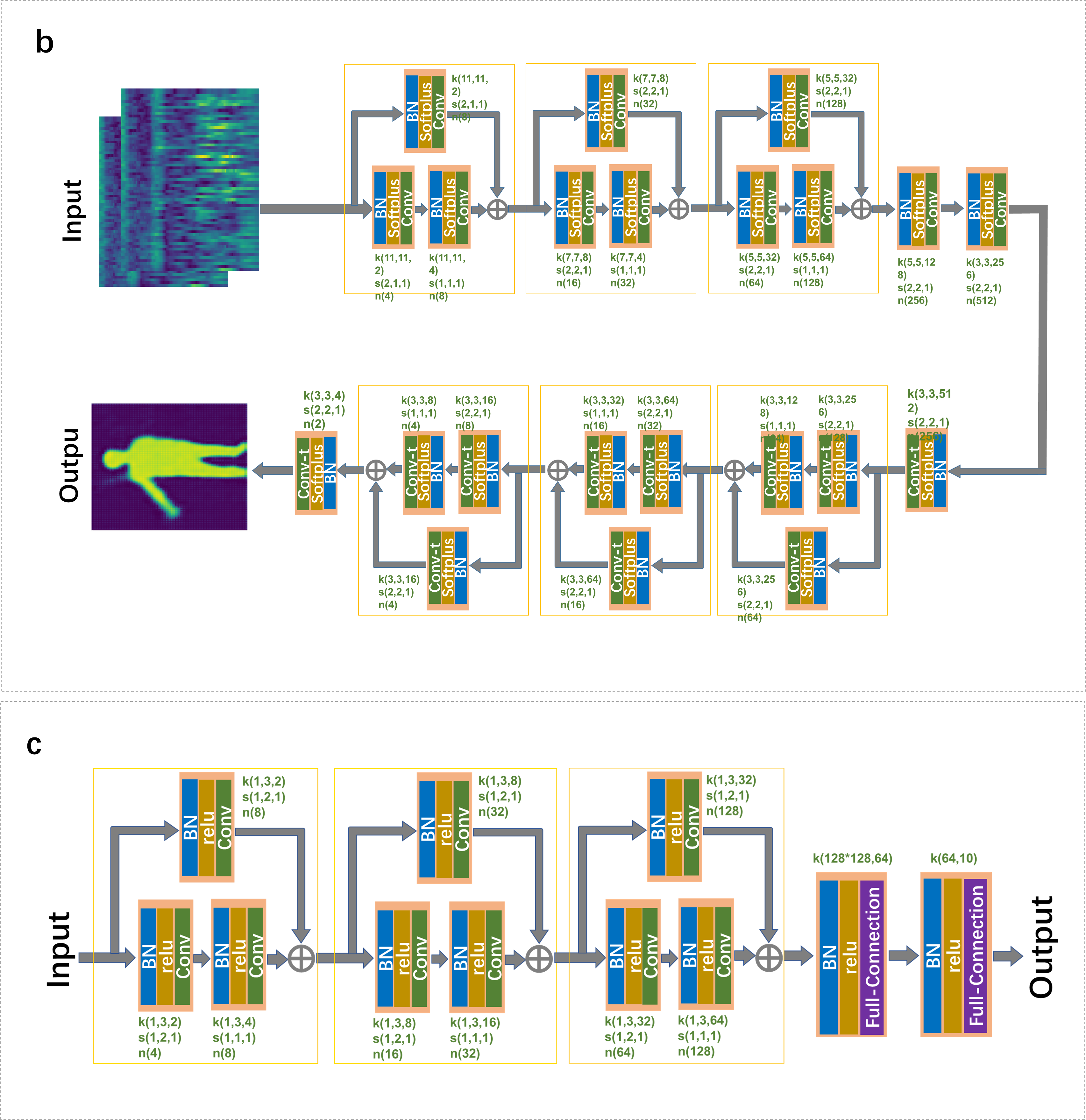

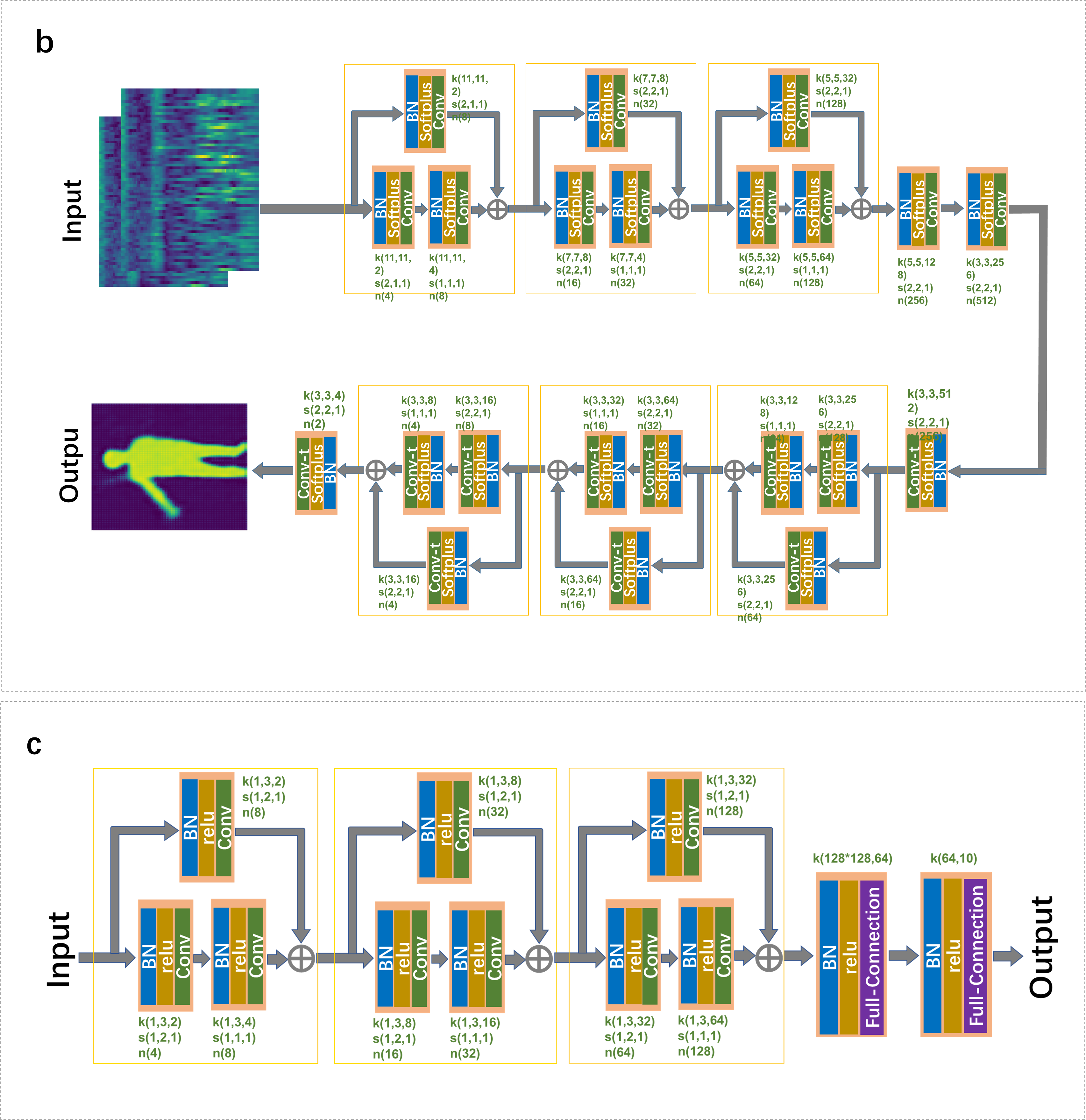


**Supplementary Figure 3 | Designed artificial neural networks and their training scheme. (a)** The supervised training procedure of IM-CNN-1. In our implementation, we use a 4-megapixel digital optical camera to achieve the required labeled training samples. Considering that the reflectivity of human body is approximated to be uniform over the to be over the whole undergoing frequencies from 2.4GHz to 2.5GHz, the color optical images are processed with binarization process as our labeled training samples. (**b**)-(**c**) are the architectures of proposed IM-CNN-1 and IM-CNN-2, in which BN denotes the batch normalization, softmax denotes soft-max nonlinear activation function, relu denotes ReLu activation function, $k\left( a,b,c \right)$ denotes the convolutional kernel with size of $a\times b\times c$, and $n\left( a \right)$ denotes the number of convolutional kernels as a, CNN-t denotes transpose operation of CNN.


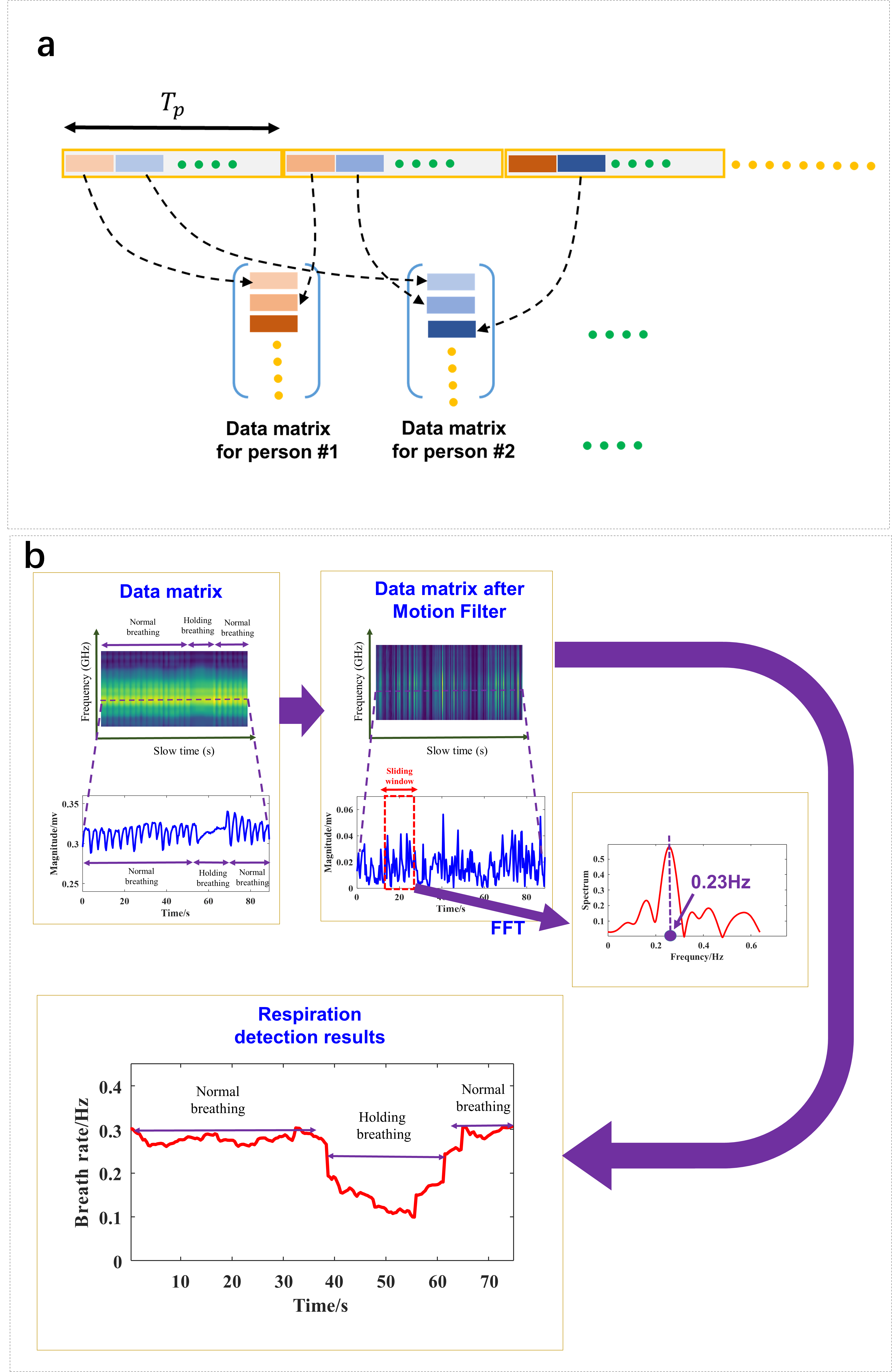


**Supplementary Figure 4 | The algorithm principle of recognitions of hand signs and vital signs. (a)** The illustrative map of respiration detection of multiple non-cooperative subject persons. **(b)** The algorithm procedure of respiration detection for one subject. The proposed algorithm consists of four steps, i.e., forming a matrix data with size of $N_{b}\times N_{d}$, performing the motion filter to remove unwanted background signals, performing the standard short-time FFT by sliding a time window along the slow time domain, and finding the respiration rate.

**
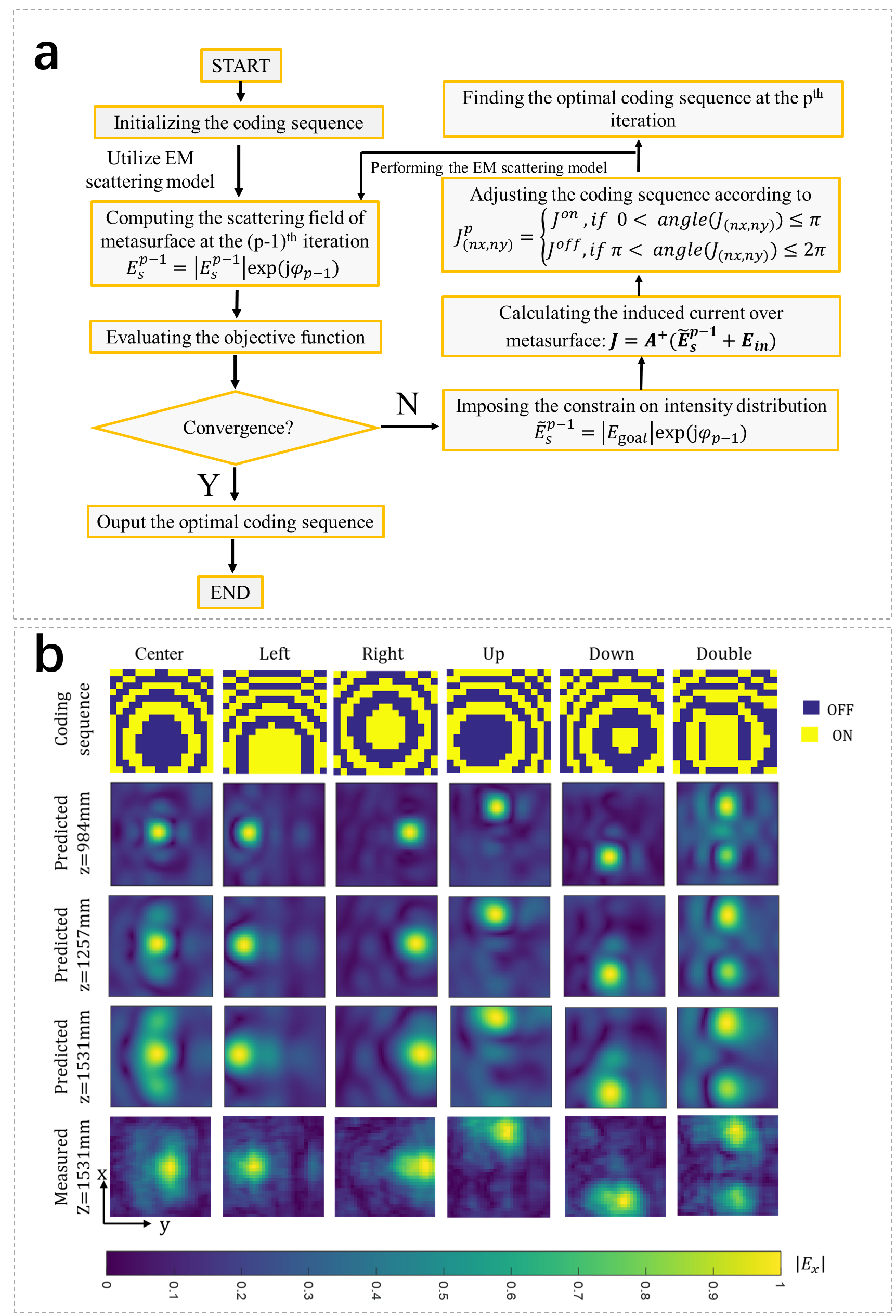
**

**Supplementary Figure 5 | Optimization scheme of metasurface for manipulating Wi-Fi signals and associated results. (a)** The flowchart of the proposed optimization algorithm for finding the desirable coding sequence of the programmable metasurface. **(b)** Selected results of spatial distribution of stray Wi-Fi signals after being focused at different desirable spots. The first row is the optimized coding patterns of programmable metasurface, the second to fourth rows are the spatial intensity distributions on z=1.0 m, z=1.3 m, and z=1.5m away from the front side of programmable metasurface using the proposed method; and the fifth row is the measured spatial intensity distributions at z=1.5m using the near-field scanning technology.


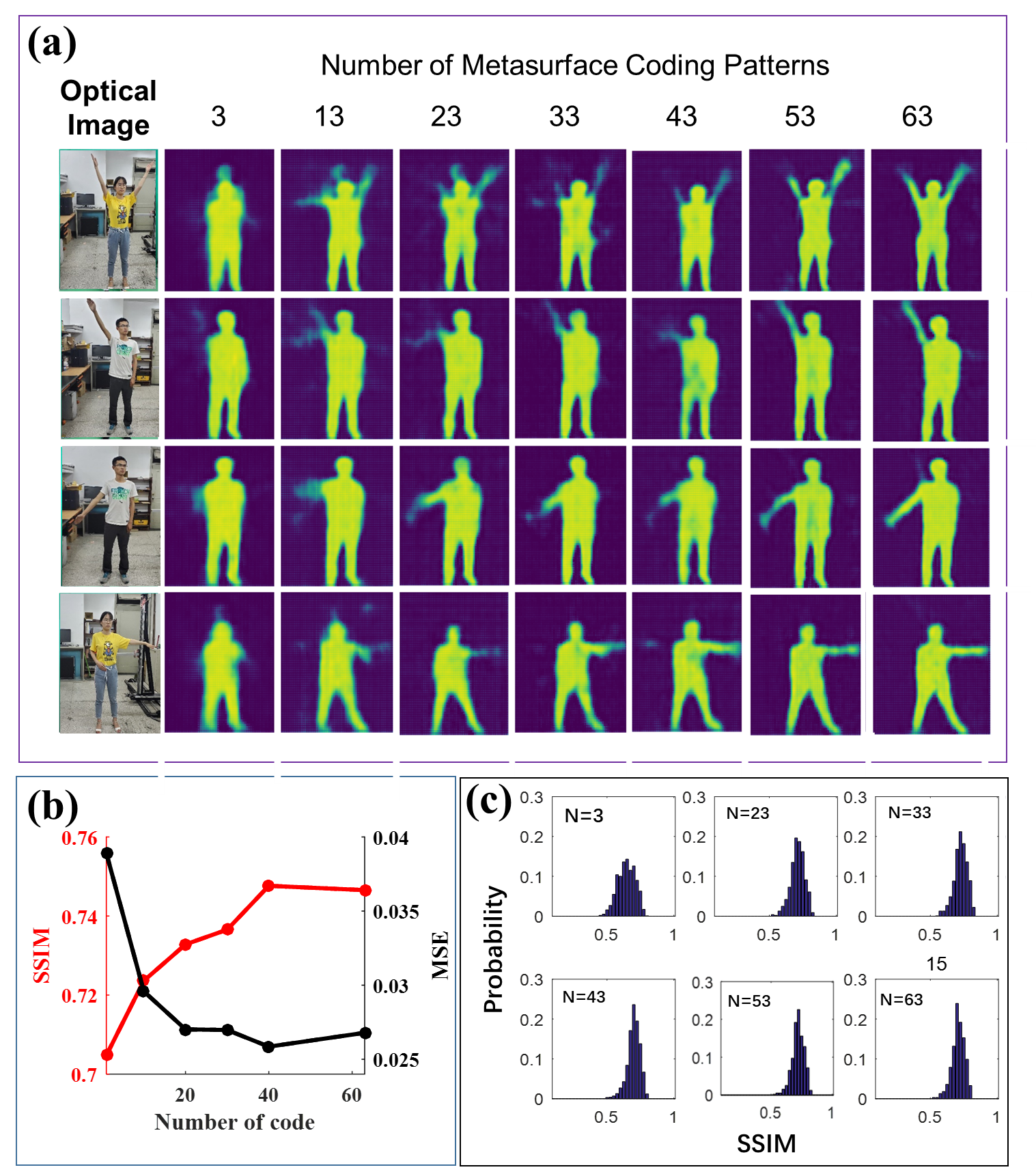


**Supplementary Figure 6 |** **The image quality of in-situ high-resolution imaging with the different numbers of coding patterns of programmable metasurface**. **(a)** Selected samples of images for different numbers of coding patterns: 3, 13, 23, 33, 43, 53, and 63. **(b)** The SSIMs and SMEs as functions of the number of coding patterns. **(c)** The statistical analysis of image quality in terms of SSIM. It can be observed that the image quality is stably improved by increasing the number of coding patterns.


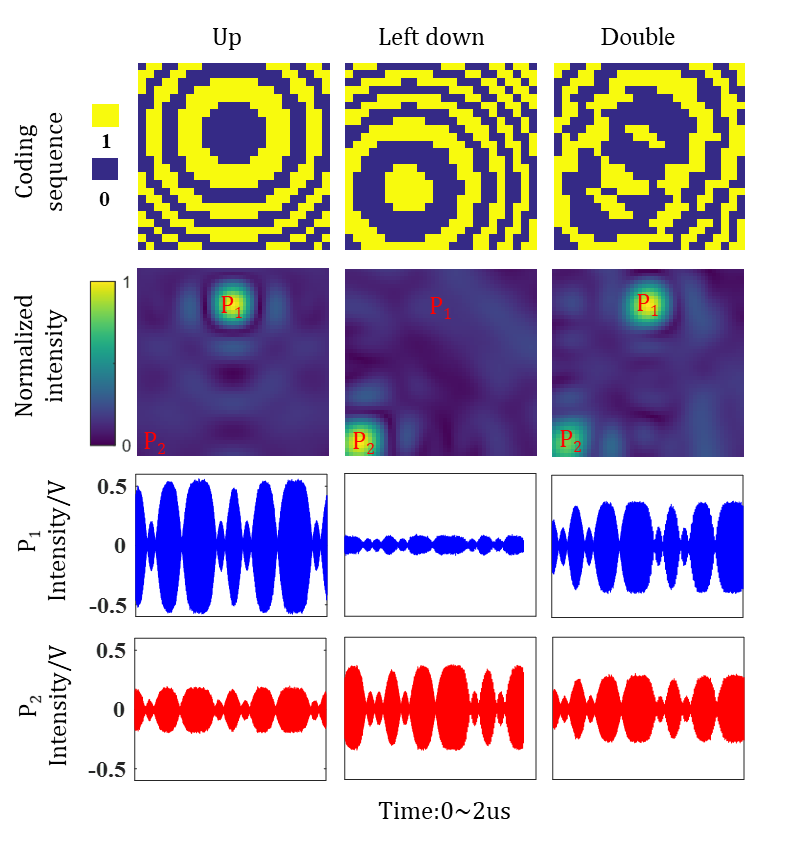


**Supplementary Figure 7 |** **Experimental results of stray Wi-Fi signals after being focused using the proposed programmable metasurface.** The first row is the optimized coding patterns of programmable metasurface. The second row is the corresponding normalized spatial intensity distributions predicted at the plane of z=1.995m away from the front side of metasurface. The third to fourth rows are the measured Wi-Fi signals at different points P1 and P2.

**Supplementary Figure 8 |** **The comparison of the SNRs of commodity Wi-Fi signals with or without being focused at the desirable spots via the programmable metasurface**. Note that after being focused, the SNR of Wi-Fi signals at the desirable spot can be remarkably enhanced with a factor of more than 20dB at around 2.4GHz.

**Supplementary Video 1. Monitoring people behind a wood wall using the proposed intelligent metasurface in active mode.**

**Supplementary Video 2. Monitoring people behind a wood wall using the proposed intelligent metasurface in passive mode.**
